# Rab2A modulates liver fibroblast growth factor 21 (FGF21) expression and systemic metabolism *via* apolipoprotein B–CREBH signaling

**DOI:** 10.1016/j.jbc.2025.110977

**Published:** 2025-11-26

**Authors:** Zi-yue Chen, Min Xu, Shuai Chen, Liang Chen

**Affiliations:** 1 State Key Laboratory of Pharmaceutical Biotechnology and MOE Key Laboratory of Model Animal for Disease Study, Model Animal Research Center, School of Medicine, Nanjing University, Nanjing, China; 2 Institute of Trauma and Metabolism, Zhengzhou Central Hospital, Zhengzhou University, Zhengzhou, China; 3 State Key Laboratory of Metabolic Dysregulation & the Prevention and Treatment of Esophageal Cancer, Tianjian Laboratory for Advanced Biomedical Sciences, Academy of Medical Sciences, Zhengzhou Central Hospital, Zhengzhou University, Zhengzhou, China; 4 College of Life Sciences, Anhui Medical University, Hefei, China

**Keywords:** nutrient sensing, AMPK, Rab2A, VLDL, CREBH, FGF21

## Abstract

Fibroblast growth factor 21 (FGF21) is a hepatokine that regulates systemic metabolism. Here, we delineate a novel regulatory pathway for FGF21 orchestrated by the small GTPase Rab2A. Our previous findings demonstrated that liver-specific deficiency of Rab2A impairs very low–density lipoprotein lipidation and promotes apolipoprotein B (APOB) accumulation. We now show that accumulated APOB drives the cleavage and activation of cAMP-responsive element–binding protein H (CREBH), a key hepatic transcription factor for FGF21 expression. Mechanistically, hepatic Rab2A inhibition protected mice from high-fat diet–induced obesity and was associated with markedly elevated circulating FGF21, the phenotype largely rescued by adenovirus-mediated knockdown of either CREBH or APOB. Collectively, we define a Rab2A–APOB–CREBH axis that is potentially essential for the hepatic regulation of FGF21.

Fibroblast growth factor 21 (FGF21) is a hepatokine predominantly synthesized in the liver, with lower expression levels in adipose tissue, skeletal muscle, and pancreas (1). Its production is highly responsive to nutritional status, being induced by starvation, protein restriction, and ketogenic diets (2, 3). Extensive research has established FGF21 as an important regulator of systemic metabolism, positioning it as a promising therapeutic target for metabolic syndromes, a notion supported by ongoing clinical trials (4, 5).

In both mice and humans, prolonged fasting robustly induces hepatic FGF21 expression (6). This induction is potentially mediated by a complex regulatory network involving several factors, including peroxisome proliferator–activated receptor α (PPARα) (7), cAMP-responsive element–binding protein H (CREBH) (8) and other signaling molecules (9, 10, 11, 12, 13). However, the precise mechanisms by which mammals sense nutrient levels to regulate FGF21 expression remain incompletely understood.

AMP-activated protein kinase (AMPK) functions as a central nutrient and energy sensor that coordinates systemic metabolism through phosphorylation of diverse substrates. Recent studies identified Rab2A, a small GTPase, as a downstream effector of AMPK signaling (14, 15). Our previous work demonstrated that hepatic Rab2A, which localizes to the Golgi apparatus, facilitates Golgi–lipid droplet contacts and is essential for very low–density lipoprotein (VLDL) lipidation and maturation (15). In this study, we identify a novel signaling axis that links nutrient sensing to FGF21 regulation. Using genetic mouse models, we demonstrate that Rab2A modulates FGF21 expression through an AMPK–Rab2A–APOB–CREBH cascade. Blocking the pathway with hepatic Rab2A deficiency leads to apolipoprotein B (APOB) accumulation in the Golgi apparatus and then promotes the cleavage and activation of the transcription factor CREBH, ultimately driving FGF21 expression and its metabolic effects.

## Results

### Hepatic Rab2A deficiency protects against high-fat diet–induced obesity

**Figure 1:**
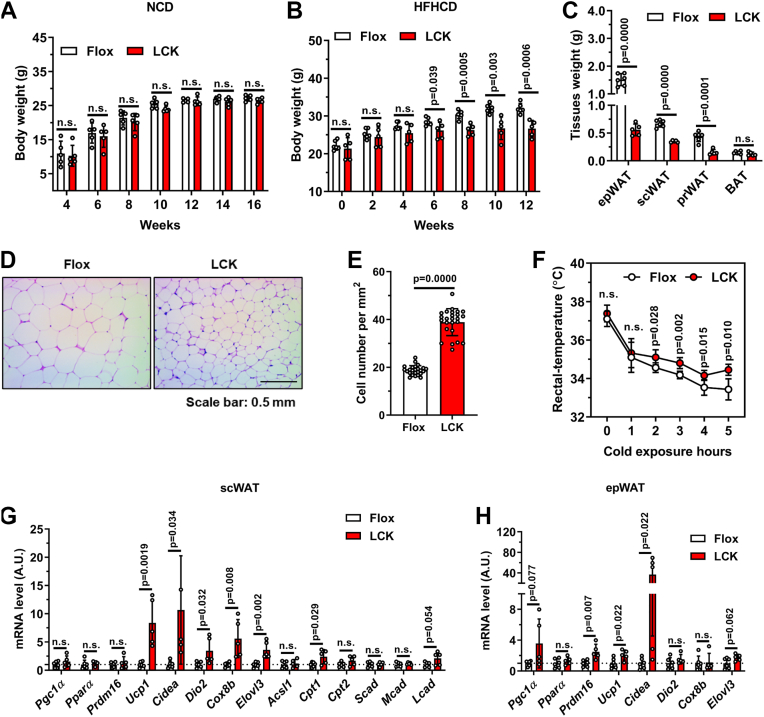
**Hepatic Rab2A deficiency protects mice from high-fat diet–induced obesity**. *A*, body weights of Flox and LCK mice fed a normal chow diet (male, n = 5 per group). *B*–*H*, flox and LCK mice were fed a high-fat–high-cholesterol diet (HFHCD) from 6 weeks of age for 12 weeks, followed by subsequent experiments (male, n = 6 *versus* 5). The curve of body weights (*B*), tissue weights of epididymal white adipose tissue (epWAT), subcutaneous white adipose tissue (scWAT), perirenal white adipose tissue (prWAT), and brown adipose tissue (BAT) (*C*), representative H&E-stained sections of epWAT (*D*) and quantitative results (*E*), rectal temperature (*F*) and mRNA expression of thermogenic-related genes in scWAT (*G*) and epWAT (*H*) were detected individually. Data are presented as mean ± SD. *p* Values were determined using the unpaired two-tailed Student’s *t* test. ns indicates no significant difference (*p* > 0.05). LCK, liver-specific Rab2A knockout.

Building on our previous work demonstrating a role for hepatic Rab2A in lipoprotein lipidation (15), we further investigated its potential impact on systemic energy homeostasis. While liver-specific Rab2A knockout (LCK) mice showed normal body weight on a chow diet (Fig. 1*A*), they exhibited significant resistance to high-fat–high-cholesterol diet (HFHCD)–induced weight gain compared with Flox control littermates (Fig. 1*B*). This phenotype was associated with a reduction in the mass of several adipose depots, including epididymal (epWAT), subcutaneous (scWAT), and perirenal white adipose tissues (Fig. 1*C*), alongside smaller adipocyte size (Fig. 1, *D* and *E*).

To explore the mechanism underlying this metabolic resistance, we assessed thermogenic capacity. LCK mice maintained a higher rectal temperature upon cold exposure (Fig. 1*F*) and showed elevated expression of thermogenic genes, including *Ucp1* and *Cidea*, in both scWAT and epWAT (Fig. 1, *G* and *H*). Collectively, these results demonstrate that hepatic Rab2A deficiency enhances thermogenic gene expression in adipose tissue and protects against diet-induced obesity.

### Hepatic Rab2A inhibition increases FGF21 expression and secretion

**Figure 2:**
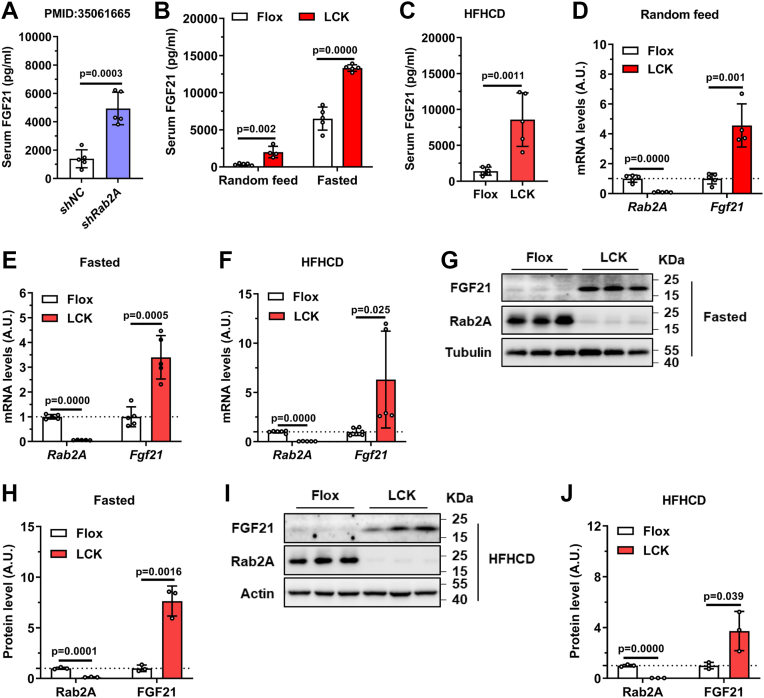
**Liver-specific inhibition of Rab2A increases FGF21 expression and secretion**. *A*, serum FGF21 levels in *shNC* and *shRab2A* mice (male, n = 5 per group; samples from PMID: 35061665). *B*, serum FGF21 levels in Flox and LCK mice under “Random feed” and “Fasted” conditions (male, n = 5 per group). *C*, serum FGF21 levels in Flox and LCK mice after 12 weeks of HFHCD feeding (male, n = 6 *versus* 5). *D*, hepatic *Fgf21* mRNA levels in “Random feed” Flox and LCK mice at 10 weeks of age (male, n = 5 per group). *E*, hepatic *Fgf21* mRNA levels in “Fasted” Flox and LCK mice at 16 weeks of age (male, n = 5 per group). *F*, hepatic *Fgf21* mRNA levels in Flox and LCK mice after 12 weeks of HFHCD feeding (male, n = 6 *versus* 5). *G*, representative immunoblots of hepatic FGF21 protein in “Fasted” Flox and LCK mice at 16 weeks of age. *H*, quantification of the immunoblots in (*G*). *I*, representative immunoblots of hepatic FGF21 protein in Flox and LCK mice after 12 weeks of HFHCD feeding. *J*, quantification of the immunoblots in (*I*). Data are presented as mean ± SD. *p* Values were determined using the unpaired two-tailed Student’s *t* test. FGF21, fibroblast growth factor 21; HFHCD, high-fat–high-cholesterol diet; LCK, liver-specific Rab2A knockout.

To elucidate the mechanism by which hepatic Rab2A regulates thermogenesis, we focused on FGF21, a hepatokine known to promote energy expenditure (16, 17, 18). This direction was prompted by our previous findings that adeno-associated virus (AAV)–mediated Rab2A knockdown markedly upregulates hepatic *Fgf21* mRNA level (14). Consistently, serum FGF21 levels were significantly increased in *shRab2A* mice than in controls (Fig. 2*A*). We further confirmed this effect in LCK mice across various nutritional conditions: “Random feed,” “Fasted,” and “HFHCD” LCK mice all exhibited elevated serum FGF21 levels (Fig. 2, *B* and *C*) and increased hepatic *Fgf21* mRNA expression levels (Fig. 2, *D*–*F*). Accordingly, immunoblot analysis demonstrated that hepatic FGF21 protein levels were also higher in LCK mice under “Fasted” (Fig. 2, *G* and *H*) and “HFHCD” (Fig. 2, *I* and *J*) conditions. Together, these results establish hepatic Rab2A as a key regulator of FGF21 expression and secretion.

### Hepatic Rab2A deficiency promotes CREBH cleavage and activation

**Figure 3:**
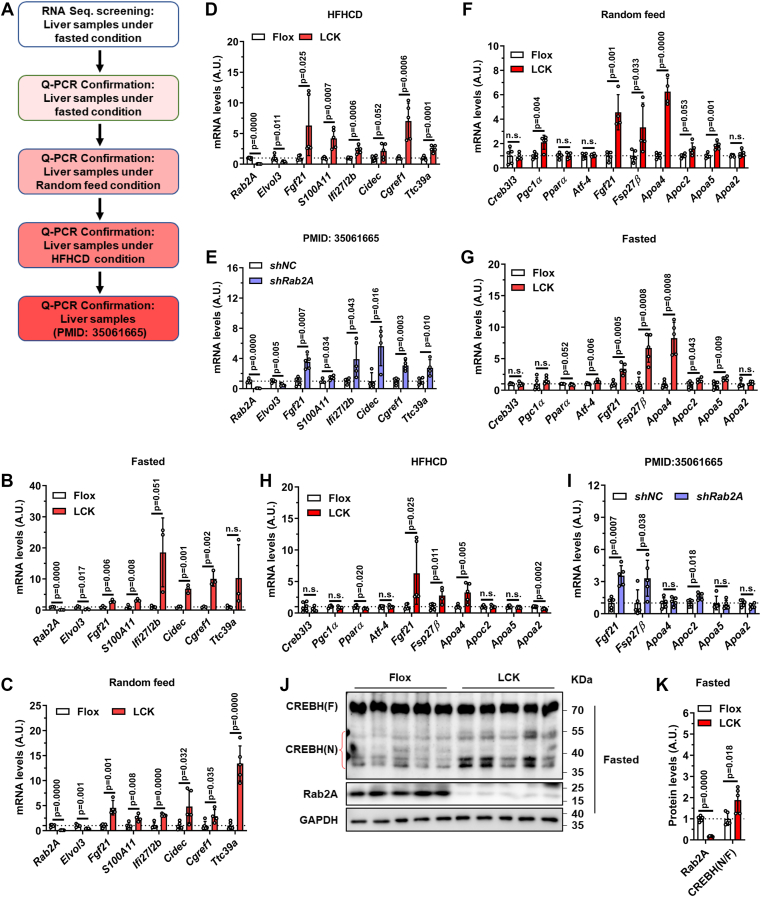
**Hepatic Rab2A deficiency promotes CREBH cleavage and activation**. *A*, the screening flow diagram. *B*–*E*, quantitative PCR validation of the RNA sequencing results in the liver samples from: “Fasted” Flox and LCK mice at 16 weeks of age (male, n = 3 per group) (*B*), “Random feed” Flox and LCK mice at 10 weeks of age (male, n = 5 per group) (*C*), Flox and LCK mice after 12 weeks of HFHCD feeding (male, n = 6 *versus* 5) (*D*) and *shNC* and *shRab2A* mice (male, n = 5 per group; samples from PMID: 35061665) (*E*). *F*–*I*, quantification of the mRNA levels including *Creb3l3* and its downstream-regulated genes in the liver samples from “Random feed” Flox and LCK mice at 10 weeks of age (male, n = 5 per group) (*F*), “Fasted” Flox and LCK mice at 16 weeks of age (male, n = 3 per group) (*G*), Flox and LCK mice after 12 weeks of HFHCD feeding (male, n = 6 *versus* 5) (*H*) and *shNC* and *shRab2A* mice (male, n = 5 per group; samples from PMID: 35061665) (*I*). *J*, representative immunoblots of hepatic CREBH cleavage in “Fasted” Flox and LCK mice at 16 weeks of age. *K*, quantification of the immunoblots in (*J*). Data are presented as mean ± SD. *p* Values were determined using the unpaired two-tailed Student’s *t* test. ns indicates no significant difference (*p* > 0.05). CREBH, cAMP-responsive element–binding protein H; HFHCD, high-fat–high-cholesterol diet; LCK, liver-specific Rab2A knockout.

To identify the regulatory network driving FGF21 upregulation in LCK mice, we performed RNA sequencing in the livers from fasted mice, followed by quantitative PCR (qPCR) validation (Fig. 3*A*). Screening with a threshold of fold change >2 and *p* < 0.01 identified 175 differentially expressed genes (Table S1). Subsequent validation across multiple conditions of Rab2A inhibition revealed only seven consistently upregulated genes, including *Fgf21* and *Fsp27β/Cidec* (Fig. 3, *B*–*E*).

The transcription factor CREBH, which is nutritionally regulated and known to control *Fgf21* and *Fsp27β* expression, emerged as a strong candidate (8, 19). Notably, this pattern specifically implicated CREBH rather than PPARα, another established regulator of *Fgf21* and genes involved in fatty acid oxidation and ketogenesis. We confirmed CREBH activation through elevated expression of its canonical targets (*Apoa4*, *Fgf21*, and *Fsp27β*) in Rab2A-deficient livers under all conditions tested (Fig. 3, *F*–*I*). Moreover, immunoblot analysis demonstrated significantly enhanced CREBH proteolytic cleavage—the key activation step—in fasted LCK mice (Fig. 3, *J* and *K*). In summary, these results indicate that increased FGF21 expression in the liver of Rab2A-deficient mice is mediated primarily by CREBH activation.

### CREBH knockdown reverses FGF21 induction and metabolic phenotypes in LCK mice

**Figure 4:**
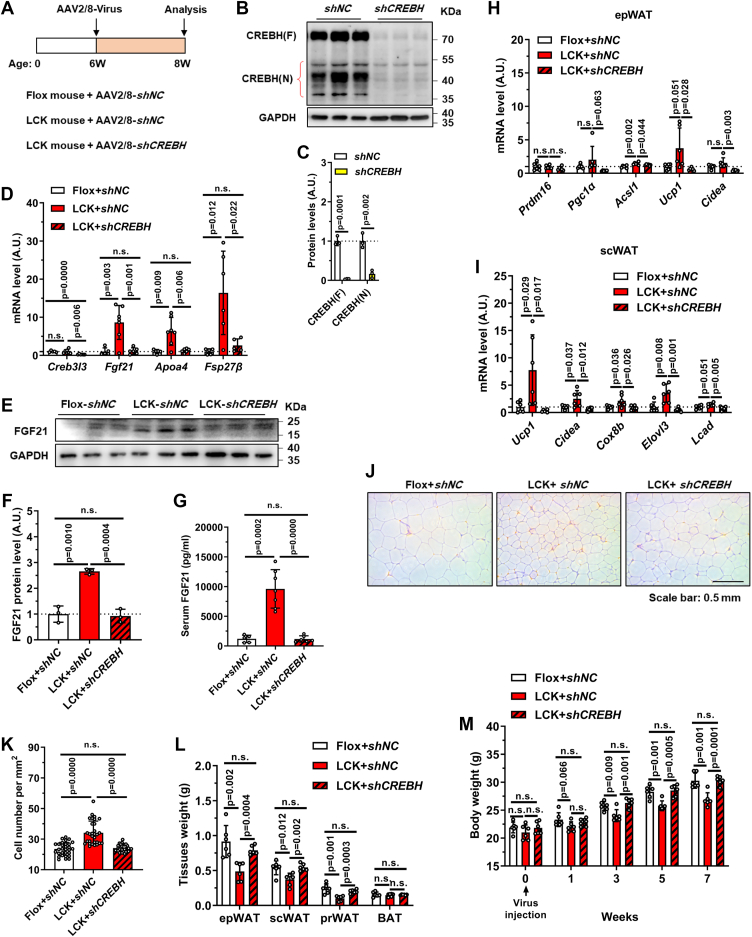
**CREBH knockdown rescues FGF21 expression and the metabolic phenotypes of LCK mice**. *A*, schematic of the experimental timeline for adeno-associated virus (AAV) injection in Flox and LCK mice. *B* and *C*, representative immunoblots and quantification of hepatic CREBH protein levels after AAV-mediated knockdown. *D*–*M*, analyses performed after 7 weeks of AAV injection and subsequent euthanasia (male, n = 6 *versus* 6 *versus* 6): Hepatic mRNA levels of *Creb3l3*, *Fgf21*, *Apoa4*, and *Fsp27β* (*D*). Representative immunoblots and quantification of hepatic FGF21 protein levels (*E* and *F*). Serum FGF21 levels (*G*). mRNA expression of thermogenic-related genes in epWAT (*H*) and scWAT (*I*). Representative H&E-stained sections of epWAT (*J*) and quantitative results (*K*). The weights of adipose tissues and the whole body (*L* and *M*). Data are presented as mean ± SD. *p* Values were determined using the unpaired two-tailed Student’s *t* test. ns indicates no significant difference (*p* > 0.05). CREBH, cAMP-responsive element–binding protein H; epWAT, epididymal white adipose tissue; FGF21, fibroblast growth factor 21; LCK, liver-specific Rab2A knockout; scWAT, subcutaneous white adipose tissue.

To establish CREBH as the critical mediator downstream of hepatic Rab2A deficiency, we conducted a genetic rescue experiment using AAV-*shCREBH* in LCK mice (Fig. 4*A*). CREBH knockdown was initially validated by reduced protein level (Fig. 4, *B* and *C*), mRNA level (Fig. 4*D*), and suppressed expression of its transcriptional targets (Fig. 4*D*). This intervention effectively normalized the elevated FGF21 level in LCK mice, reducing it to control levels at mRNA (Fig. 4*D*), protein (Fig. 4, *E* and *F*), and circulating levels (Fig. 4*G*).

The normalization of FGF21 was accompanied by a complete reversal of metabolic phenotypes in LCK mice: *shCREBH*-treated LCK mice showed downregulation of thermogenic genes in both epWAT and scWAT (Fig. 4, *H* and *I*), increased adipocyte size (Fig. 4, *J* and *K*), and elevated mass of epWAT, scWAT, and perirenal WAT (Fig. 4*L*). Consequently, the body weight difference between LCK and control mice was abolished upon CREBH inhibition (Fig. 4*M*). These results demonstrate that CREBH activation is necessary for the increased FGF21 expression and metabolically beneficial phenotypes observed in LCK mice.

### APOB knockdown normalizes CREBH–FGF21 signaling and the metabolic phenotypes of LCK mice

**Figure 5:**
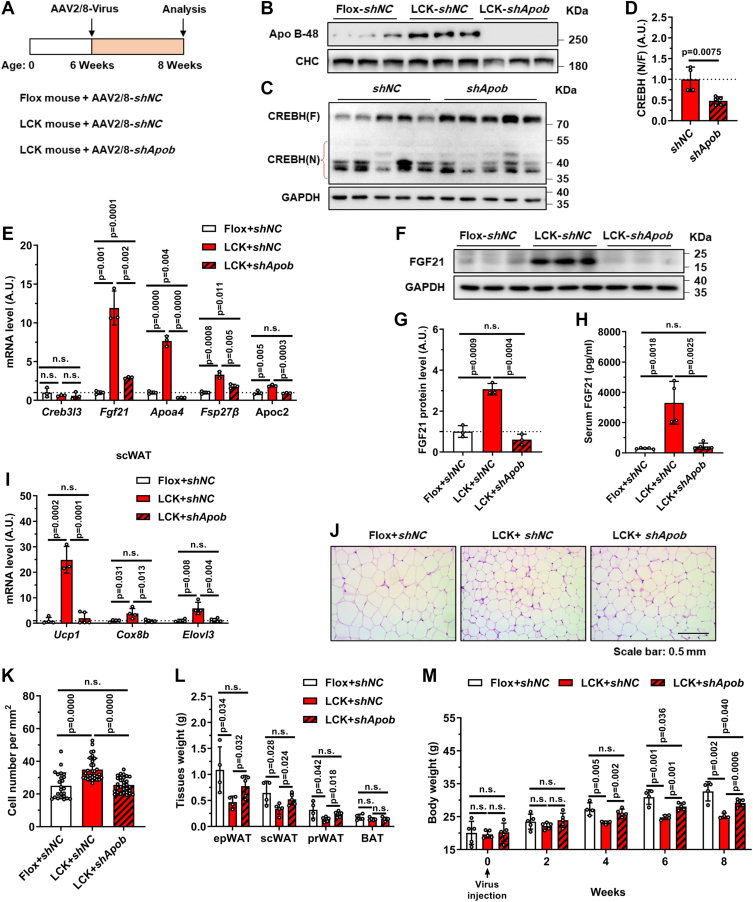
**APOB knockdown rescues CREBH activation, FGF21 expression, and the metabolic phenotypes of LCK mice**. *A*, schematic of the experimental timeline for adeno-associated virus (AAV) injection in Flox and LCK mice. *B*, representative immunoblots of hepatic APOB protein levels after AAV-mediated knockdown. *C* and *D*, representative immunoblots and quantification of hepatic CREBH cleavage levels after APOB knockdown. *E*–*M*, analyses performed after 8 weeks of AAV injection and subsequent euthanasia (male, n = 5 *versus* 5 *versus* 5): hepatic mRNA levels of *Creb3l3*, *Fgf21*, *Apoa4*, *Fsp27β*, and *Apoc2* (*E*). Representative immunoblots and quantification of hepatic FGF21 protein levels (*F* and *G*). Serum FGF21 levels (*H*). mRNA expression of thermogenic-related genes in scWAT (*I*). Representative H&E-stained sections of epWAT (*J*) and quantitative results (*K*). The weights of adipose tissues and the whole body (*L* and *M*). Data are presented as mean ± SD. *p* Values were determined using the unpaired two-tailed Student’s *t* test. ns indicates no significant difference (*p* > 0.05). AAV, adeno-associated virus; APOB, apolipoprotein B; CREBH, cAMP-responsive element–binding protein H; epWAT, epididymal white adipose tissue; FGF21, fibroblast growth factor 21; LCK, liver-specific Rab2A knockout; scWAT, subcutaneous white adipose tissue.

The mechanism linking Rab2A to CREBH activation remained unclear until a seminal study suggested a role for VLDL assembly in regulating CREBH proteolysis (20). Given our previous finding that Rab2A deficiency impairs VLDL lipidation and causes APOB accumulation (Fig. 5*A*) (15), we hypothesized that APOB may mediate CREBH activation. To test this, we performed a rescue experiment using AAV-*shApob* in LCK mice (Fig. 5*A*). Efficient APOB knockdown was confirmed by immunoblotting (Fig. 5*B*). Strikingly, APOB depletion in LCK mice substantially reduced CREBH cleavage and transcriptional activity (Fig. 5, *C*–*E*). Consequently, FGF21 expression was normalized to control levels in *shApob*-treated LCK mice, as evidenced by mRNA (Fig. 5*E*), hepatic protein (Fig. 5, *F* and *G*), and serum measurements (Fig. 5*H*).

Restoration of the CREBH–FGF21 axis mostly reversed the metabolic phenotypes of LCK mice: thermogenic gene expression in scWAT (Fig. 5*I*), adipocyte size in epWAT (Fig. 5, *J* and *K*), and mass of WATs (Fig. 5*L* were all comparable to Flox controls. Ultimately, APOB knockdown significantly normalized body weight in LCK mice, eliminating their resistance to weight gain (Fig. 5*M*). Together, these results establish APOB as the essential mediator through which Rab2A regulates CREBH-driven FGF21 expression and systemic energy balance.

### Fasting triggers CREBH–FGF21 signaling potentially through APOB trafficking defects

**Figure 6:**
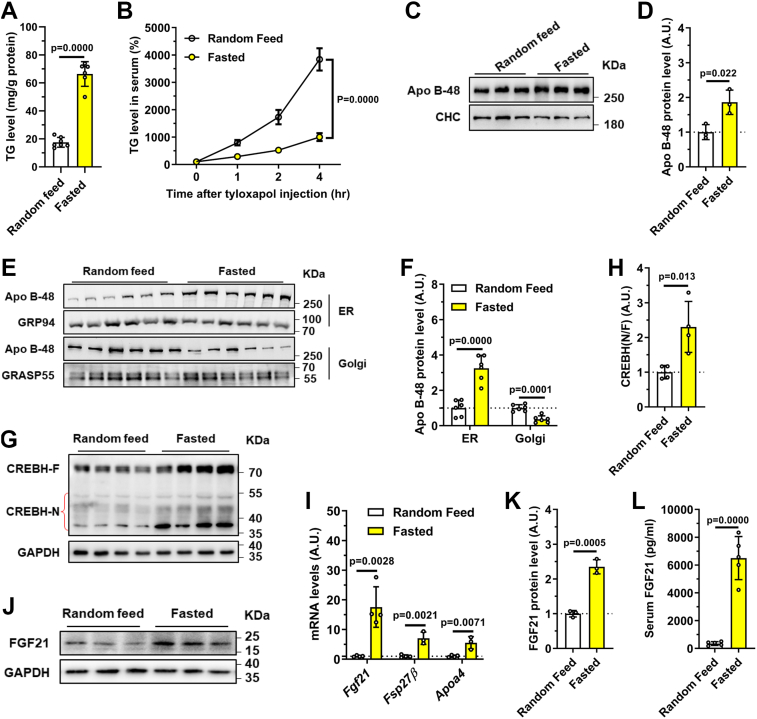
**Starvation-induced APOB trafficking defects activate the CREBH–FGF21 axis**. Wildtype C57BL/6J mice were fasted for 16 h or not prior to analysis. *A*, hepatic triglyceride (TG) levels (n = 6 per group). *B*, VLDL–TG secretion rates (n = 5 per group). *C*, representative immunoblots of total hepatic APOB. *D*, quantification of APOB protein levels from (*C*). *E*, representative immunoblots of APOB distribution in the endoplasmic reticulum (ER) and Golgi apparatus fractions. *F*, quantification of APOB levels in subcellular fractions from (*E*). *G*, representative immunoblots of CREBH cleavage. *H*, quantification of cleaved CREBH from (*G*). *I*, hepatic mRNA levels of *Fgf21*, *Fsp27β*, and *Apoa4* (n = 4 per group). *J*, representative immunoblots of hepatic FGF21 proteins. *K*, quantification of FGF21 protein levels from (*J*). *L*, serum FGF21 levels (n = 5 per group). Data are presented as mean ± SD. *p* Values were determined using the unpaired two-tailed Student’s *t* test. APOB, apolipoprotein B; CREBH, cAMP-responsive element–binding protein H; FGF21, fibroblast growth factor 21; TG, triglyceride; VLDL, very low–density lipoprotein.

While fasting potently induces the hepatic CREBH–FGF21 axis, the underlying regulatory mechanisms remain incompletely defined. To address this, we compared wildtype mice under “Random feed” and “Fasted” conditions. Fasting led to pronounced hepatic triglyceride (TG) accumulation (Fig. 6*A*), a phenotype we partially linked to fasting-activated AMPK, which suppresses Rab2A activity and thereby attenuates VLDL secretion (Fig. 6*B*) (15). In contrast to the marked APOB accumulation seen in LCK mice, fasting induced only a moderate increase in total APOB levels (Fig. 6, *C* and *D*). However, subcellular fractionation revealed a significant fasting-induced redistribution of APOB, characterized by its relative retention in the endoplasmic reticulum (ER) and reduced presence in the Golgi apparatus (Fig. 6, *E* and *F*). This disrupted ER-to-Golgi trafficking of APOB was associated with robust activation of the CREBH–FGF21 axis, evidenced by enhanced CREBH cleavage (Fig. 6, *G* and *H*), elevated hepatic FGF21 expression (Fig. 6, *I*–*K*), and increased serum FGF21 levels (Fig. 6*L*). These findings establish a link between nutrient-sensitive APOB transport and CREBH-driven FGF21 production, providing a mechanistic basis for the metabolic adaptation to fasting.

## Discussion

AMPK serves as a central sensor that coordinates nutritional and energetic status with downstream metabolic pathways. Here, we identify Rab2A as a downstream effector of AMPK that governs systemic metabolism through the CREBH–FGF21 signaling axis. Together with our previous work establishing the role of Rab2A in hepatic and serum lipid regulation (14, 15), these findings position the AMPK–Rab2A pathway as a key metabolic regulator, though the specific GTPase-activating proteins involved remain to be identified.

Hepatic CREBH serves as a key metabolic regulator that coordinates lipid homeostasis across the liver, systemic circulation, and adipose tissue, primarily through the transcriptional control of downstream genes, such as *Fgf21*, *Fsp27β*, and *Apoa4* (19, 21, 22, 23). The regulation of CREBH during fasting involves multiple intersecting pathways. While initial studies suggested that ER stress induces CREBH proteolysis (24), subsequent work demonstrated limited CREBH activation under ER stress conditions (25). Interestingly, fasting suppresses ER-associated degradation through downregulation of core components, including Sel1L and Hrd1, thereby stabilizing the ER-resident CREBH pool (26, 27). Additional regulatory mechanisms include fasting-induced CREBH acetylation, which enhances its interaction with PPARα to drive target gene expression (28).

**Figure 7:**
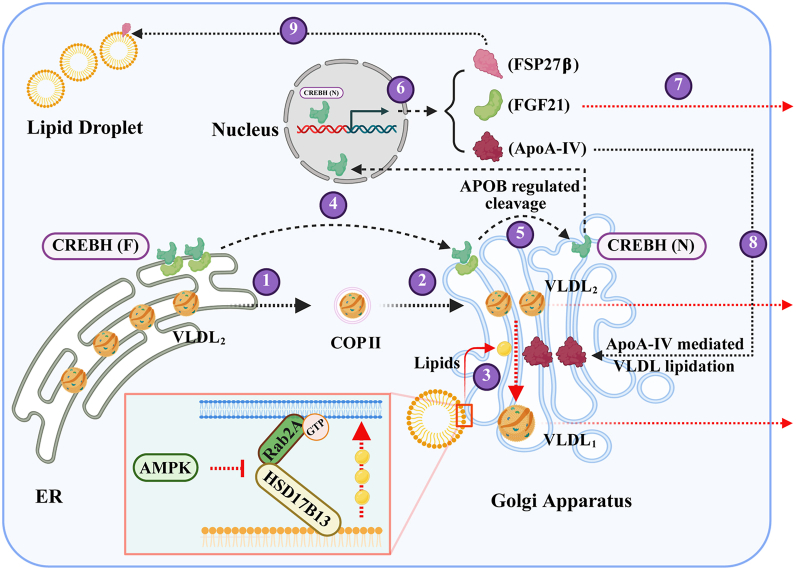
**Summary of results and work model**. A graphical abstract was created to summarize the key findings of this study, outlining a multistep regulatory pathway. VLDL assembly and maturation (steps 1–3): newly assembled VLDL_2_ particles are transported from the ER to the Golgi *via* COPII vesicles. At the Golgi, the Rab2A–HSD17B13 complex mediates lipid droplet–Golgi contacts, facilitating lipid transfer to support VLDL_1_ maturation. CREBH activation and signaling (steps 4–6): full-length CREBH resides in the ER and is transported to the Golgi under specific conditions, where its proteolytic cleavage—partially dependent on APOB availability—generates the active N-terminal fragment. This fragment translocates to the nucleus and drives transcription of *Fsp27β*, *Fgf21*, and *Apoa4*. Systemic metabolic regulation (steps 7–9): secreted FGF21 promotes adipose tissue lipolysis; ApoA-IV enhances VLDL lipidation and expansion in the Golgi; and lipid droplet–localized FSP27β facilitates hepatic lipid storage. Pathophysiological perturbations: in Rab2A-deficient hepatocytes, disrupted lipid droplet–Golgi contacts reduce lipid flux to the Golgi, impairing VLDL maturation. This leads to APOB accumulation in the Golgi, which promotes CREBH cleavage and elevates FGF21 expression. During fasting, AMPK-mediated suppression of Rab2A activity, combined with partial inhibition of APOB ER-to-Golgi transport, further reduces VLDL secretion. These changes are sensed by CREBH, enhancing its activation and driving a transcriptional program that adaptively regulates systemic lipid homeostasis. Graphical abstract created with BioRender (Chen, 2025; https://BioRender.com/7gyj0x2). AMPK, AMP-activated protein kinase; APOB, apolipoprotein B; COPII, coat protein complex II; CREBH, cAMP-responsive element–binding protein H; ER, endoplasmic reticulum; VLDL, very low–density lipoprotein.

Previous work demonstrated that blocking VLDL assembly *via* knockdown of microsomal TG transfer protein or APOB elevates hepatic TGs yet paradoxically attenuates CREBH cleavage (20). Building on our current findings, we propose that APOB potentially represents a novel physiological sensor or regulator of CREBH activation. We hypothesize that fasting disrupts hepatic lipid homeostasis and impairs APOB trafficking, triggering a compensatory response wherein CREBH senses altered APOB availability through an unknown mechanism. This sensing pathway drives expression of *Fgf21*, *Fsp27β*, and *Apoa4* to restore systemic lipid homeostasis, representing an adaptive mechanism to nutritional stress that complements established regulatory pathways (Fig. 7).

## Experimental procedures

### Animals

C57BL/6J mice (strain no.: N000013), Rab2A-flox/flox mice (strain no.: T018874), and Alb-iCre mice (strain no.: T003814) were procured from Gempharmatech Co, Ltd. Mice with LCK were generated by crossing Rab2A-flox/flox mice (Flox) with Alb-iCre mice. All mice were genotyped and housed under standard conditions as previously described (15). For rescue studies in LCK mice, AAV serotype 2/8 (AAV2/8) particles expressing shRNA targeting APOB (15) or CREBH were administered. The targeting sequence for CREBH was 5′-GGACATAGCGGCTGGAAAGAT-3′.

Generally, the serum and tissues were collected for analysis under the indicated experimental conditions. Circulating levels of FGF21 were measured using ELISA kits (R&D Systems; MF2100). Body weight, tissue mass, and body temperature were monitored. Mice were maintained in a specific pathogen-free facility on a 12-h light–dark cycle with ad libitum access to food and water. All animal experiments were approved by the Ethics Committees of Anhui Medical University (approval numbers: LLSC20200327 and LLSC20241078) and performed in accordance with institutional guidelines.

### Western blotting and antibodies

Western blotting was performed using standard protocols. Briefly, liver tissues were homogenized in radioimmunoprecipitation assay lysis buffer supplemented with proteinase inhibitors. Protein concentrations were determined, and equal amounts of lysate were separated by SDS-PAGE, transferred to polyvinylidene difluoride membranes, and probed with the indicated primary antibodies followed by horseradish peroxidase (HRP)–conjugated secondary antibodies. Signals were captured using an autoradiography machine (Tanon-5200). Band intensities were quantified using ImageJ (National Institutes of Health, https://imagej.nih.gov/ij/) and normalized to loading controls. The following primary and secondary antibodies were used: goat anti FGF21 (R&D Systems; AF3057), mouse anti-Rab2A (Proteintech; 67501-1-Ig), mouse anti-tubulin (DUONENG-BIO; AB0178801), mouse anti-actin (Zen-Bioscience; 200068-8F10), rabbit anti-CREBH (Kerafast; EWS101), rabbit anti-GAPDH (Proteintech; 10494-1-AP), rabbit anti-APOB (Proteintech; 20578-1-AP), mouse anti-CHC (Santa Cruz; sc-12734), rabbit anti-GRP94 (Proteintech; 14700-1-AP), mouse anti-GRASP55 (Santa Cruz; sc-271840), HRP-conjugated goat anti-mouse IgG (Jackson ImmunoResearch; 115-035-003), HRP-conjugated goat anti-rabbit IgG (Jackson ImmunoResearch; 111-035-003), and HRP-conjugated rabbit anti-goat IgG (Jackson ImmunoResearch; 305-035-003).

### RNA isolation, RNA sequencing, and qPCR

Total RNA was extracted from liver samples using RNAiso Plus reagent (Takara; 9109) according to the manufacturer's instructions. Following quality control, 1 μg of RNA was reverse transcribed into complementary DNA using HiScript III RT SuperMix (+gDNA wiper) (Vazyme Biotechnology; R323-01). RNA sequencing libraries were prepared and analyzed as previously described (14). qPCR was performed using Taq SYBR Green qPCR Premix (EG20117M; Yugong Biotech) on an Applied Biosystems StepOnePlus Real-Time PCR system. The results of RNA sequencing and primer sequences used are listed in Table S1.

### Histology and imaging

Histology and imaging were carried out as previously described (29). Briefly, the adipose tissues were fixed, embedded, and sectioned by a Leica RM2016 microtome. The slices were stained with hematoxylin and eosin and imaged with a phase-contrast microscope (Leica DMi1). The cell number was quantified from acquired images.

### VLDL–TG secretion

Mice were tail vein injected with tyloxapol at a dose of 500 mg/kg following a 16-h fasting or not. Then, the tail vein serum was collected at 0, 1, 2, and 4 h, individually, and subjected to TG and cholesterol detection.

### TG level detection in the liver

Hepatic TG levels were quantified from frozen liver samples. Briefly, weighed tissue was saponified in ethanolic KOH. Glycerol was then extracted with ethanol and MgCl_2_, and its level was determined with a free glycerol reagent (F6428; Sigma–Aldrich), using glycerol (G7793; Sigma–Aldrich) as the standard for calculation.

### Subcellular fractionation of hepatic ER and Golgi apparatus

Subcellular fractionation of the ER and Golgi apparatus from liver tissues was performed as described previously (15, 30). The fractionated samples and quality control data for these experiments were derived from our prior study (15).

### Statistical analysis

Data are presented as mean ± SD. Statistical analyses were performed using GraphPad Prism 7 software (GraphPad Software, Inc). For comparisons between two groups, an unpaired, two-tailed Student’s *t* test was used. For analyses involving multiple factors or time courses, two-way ANOVA was applied. A *p* value of less than 0.05 was considered statistically significant.

## Data availability

All data are included in this article and the supporting information.

## Supporting information

This article contains supporting information.

## Conflict of interest

The authors declare that they have no conflicts of interest with the contents of this article.

## References

[1] Zhang X., Li Z., Wang S., Chen Y. (2025). Distinct Fgf21 expression patterns in various tissues in response to different dietary regimens using a reporter mouse model. Nutrients.

[2] Martinez-Garza U., Torres-Oteros D., Yarritu-Gallego A., Marrero P.F., Haro D., Relat J. (2019). Fibroblast growth factor 21 and the adaptive response to nutritional challenges. Int. J. Mol. Sci..

[3] Solon-Biet S.M., Cogger V.C., Pulpitel T., Heblinski M., Wahl D., McMahon A.C. (2016). Defining the nutritional and metabolic context of FGF21 using the geometric framework. Cell Metab..

[4] Geng L., Lam K.S.L., Xu A. (2020). The therapeutic potential of FGF21 in metabolic diseases: from bench to clinic. Nat. Rev. Endocrinol..

[5] Harrison S.A., Rolph T., Knott M., Dubourg J. (2024). FGF21 agonists: an emerging therapeutic for metabolic dysfunction-associated steatohepatitis and beyond. J. Hepatol..

[6] Fazeli P.K., Lun M., Kim S.M., Bredella M.A., Wright S., Zhang Y. (2015). FGF21 and the late adaptive response to starvation in humans. J. Clin. Invest.

[7] Badman M.K., Pissios P., Kennedy A.R., Koukos G., Flier J.S., Maratos-Flier E. (2007). Hepatic fibroblast growth factor 21 is regulated by PPARalpha and is a key mediator of hepatic lipid metabolism in ketotic states. Cell Metab..

[8] Kim H., Mendez R., Zheng Z., Chang L., Cai J., Zhang R. (2014). Liver-enriched transcription factor CREBH interacts with peroxisome proliferator-activated receptor alpha to regulate metabolic hormone FGF21. Endocrinology.

[9] Byun S., Seok S., Kim Y.C., Zhang Y., Yau P., Iwamori N. (2020). Fasting-induced FGF21 signaling activates hepatic autophagy and lipid degradation via JMJD3 histone demethylase. Nat. Commun..

[10] Li Y., Wong K., Giles A., Jiang J., Lee J.W., Adams A.C. (2014). Hepatic SIRT1 attenuates hepatic steatosis and controls energy balance in mice by inducing fibroblast growth factor 21. Gastroenterology.

[11] Laeger T., Albarado D.C., Burke S.J., Trosclair L., Hedgepeth J.W., Berthoud H.R. (2016). Metabolic responses to dietary protein restriction require an increase in FGF21 that is delayed by the absence of GCN2. Cell Rep..

[12] Iizuka K., Takeda J., Horikawa Y. (2009). Glucose induces FGF21 mRNA expression through ChREBP activation in rat hepatocytes. FEBS Lett..

[13] Cornu M., Oppliger W., Albert V., Robitaille A.M., Trapani F., Quagliata L. (2014). Hepatic mTORC1 controls locomotor activity, body temperature, and lipid metabolism through FGF21. Proc. Natl. Acad. Sci. U. S. A..

[14] Chen Z.Y., Sun Y.T., Wang Z.M., Hong J., Xu M., Zhang F.T. (2022). Rab2A regulates the progression of nonalcoholic fatty liver disease downstream of AMPK-TBC1D1 axis by stabilizing PPARgamma. PLoS Biol..

[15] Xu M., Chen Z.Y., Li Y., Li Y., Guo G., Dai R.Z. (2024). Rab2A-mediated Golgi-lipid droplet interactions support very-low-density lipoprotein secretion in hepatocytes. EMBO J..

[16] Holland W.L., Adams A.C., Brozinick J.T., Bui H.H., Miyauchi Y., Kusminski C.M. (2013). An FGF21-adiponectin-ceramide axis controls energy expenditure and insulin action in mice. Cell Metab..

[17] Owen B.M., Ding X., Morgan D.A., Coate K.C., Bookout A.L., Rahmouni K. (2014). FGF21 acts centrally to induce sympathetic nerve activity, energy expenditure, and weight loss. Cell Metab..

[18] Gliniak C.M., Gordillo R., Youm Y.H., Lin Q., Crewe C., Zhang Z. (2025). FGF21 promotes longevity in diet-induced obesity through metabolic benefits independent of growth suppression. Cell Metab..

[19] Xu X., Park J.G., So J.S., Lee A.H. (2015). Transcriptional activation of Fsp27 by the liver-enriched transcription factor CREBH promotes lipid droplet growth and hepatic steatosis. Hepatology.

[20] Cheng D., Xu X., Simon T., Boudyguina E., Deng Z., VerHague M. (2016). Very low density lipoprotein assembly is required for cAMP-responsive element-binding protein H processing and hepatic apolipoprotein A-IV expression. J. Biol. Chem..

[21] Park J.G., Xu X., Cho S., Lee A.H. (2016). Loss of transcription factor CREBH accelerates diet-induced atherosclerosis in mice. Arteriosclerosis Thromb. Vasc. Biol..

[22] Nakagawa Y., Satoh A., Yabe S., Furusawa M., Tokushige N., Tezuka H. (2014). Hepatic CREB3L3 controls whole-body energy homeostasis and improves obesity and diabetes. Endocrinology.

[23] Lee J.H., Giannikopoulos P., Duncan S.A., Wang J., Johansen C.T., Brown J.D. (2011). The transcription factor cyclic AMP-responsive element-binding protein H regulates triglyceride metabolism. Nat. Med..

[24] Zhang K., Shen X., Wu J., Sakaki K., Saunders T., Rutkowski D.T. (2006). Endoplasmic reticulum stress activates cleavage of CREBH to induce a systemic inflammatory response. Cell.

[25] Xu X., Park J.G., So J.S., Hur K.Y., Lee A.H. (2014). Transcriptional regulation of apolipoprotein A-IV by the transcription factor CREBH. J. Lipid Res..

[26] Bhattacharya A., Sun S., Wang H., Liu M., Long Q., Yin L. (2018). Hepatic Sel1L-Hrd1 ER-associated degradation (ERAD) manages FGF21 levels and systemic metabolism via CREBH. EMBO J..

[27] Bailey D., Barreca C., O'Hare P. (2007). Trafficking of the bZIP transmembrane transcription factor CREB-H into alternate pathways of ERAD and stress-regulated intramembrane proteolysis. Traffic.

[28] Kim H., Mendez R., Chen X., Fang D., Zhang K. (2015). Lysine acetylation of CREBH regulates fasting-induced hepatic lipid metabolism. Mol. Cell Biol..

[29] Chen L., Chen Q., Xie B., Quan C., Sheng Y., Zhu S. (2016). Disruption of the AMPK-TBC1D1 nexus increases lipogenic gene expression and causes obesity in mice via promoting IGF1 secretion. Proc. Natl. Acad. Sci. U. S. A..

[30] Li X., Ye J., Zhou L., Gu W., Fisher E.A., Li P. (2012). Opposing roles of cell death-inducing DFF45-like effector B and perilipin 2 in controlling hepatic VLDL lipidation. J. Lipid Res..

